# Involvement of Fatty Acid Binding Protein 5 and PPAR*β*/*δ* in Prostate Cancer Cell Growth

**DOI:** 10.1155/2010/234629

**Published:** 2010-08-19

**Authors:** Elwin Morgan, Padmamalini Kannan-Thulasiraman, Noa Noy

**Affiliations:** Department of Pharmacology, Case Western Reserve University, 10900 Euclid Ave., Cleveland, OH 44106, USA

## Abstract

Fatty acid binding protein 5 (FABP5) delivers ligands from the cytosol directly to the nuclear receptor PPAR*β*/*δ* and thus facilitates the ligation and enhances the transcriptional activity of the receptor. We show here that expression levels of both FABP5 and PPAR*β*/*δ* are correlated with the tumorigenic potential of prostate cancer cell lines. We show further that FABP5 comprises a direct target gene for PPAR*β*/*δ* and thus the binding protein and its cognate receptor are engaged in a positive feedback loop. The observations demonstrate that, similarly to effects observed in mammary carcinomas, activation of the FABP5/PPAR*β*/*δ* pathway induces PPAR*β*/*δ* target genes involved in cell survival and growth and enhances cell proliferation and anchorage-independent growth in prostate cancer cells. Furthermore, the data show that downregulation of either FABP5 or PPAR*β*/*δ* inhibits the growth of the highly malignant prostate cancer PC3M cells. These studies suggest that the FABP5/PPAR*β*/*δ* pathway may play a general role in facilitating tumor progression and that inhibition of the pathway may comprise a novel strategy in treatment of cancer.

## 1. Introduction

The current treatment of prostate cancer relies on a combination of androgen ablation and radiation/chemotherapy but tumors often relapse in a more aggressive hormone-refractory form. There is thus a clear need for a better understanding of the molecular mechanisms involved in the malignant progression of prostate cancer which may lead to new therapies [1, 2]. 

The vitamin A metabolite retinoic acid (RA) controls multiple biological processes by virtue of its ability to regulate gene transcription. These activities are mediated by the ligand-activated transcription factors retinoic acid receptors (RARs) [3, 4] and peroxisome proliferator activated receptor *β*/*δ* (PPAR*β*/*δ*) [5, 6]. Activation of RAR by RA results in up-regulation of target genes that promote apoptosis, cell cycle arrest, and differentiation, for example, the cell cycle regulator Btg2 and the apoptotic gene caspase 9 [7, 8]. Hence, in cancers where RA activates RAR, it functions as a potent anticarcinogenic agent [9, 10]. However, target genes for the alternate RA receptor, PPAR*β*/*δ*, include genes that promote cell survival and proliferation, for example, the survival factor PDK1 and the angiogenic growth factor VEGF [11, 12] and thus, in cells in which RA activates this receptor, it promotes rather than inhibits growth [5, 13]. 

The partitioning of RA between its two receptors is regulated by two members of the family of intracellular lipid binding proteins (iLBPs): fatty acid binding protein 5 (FABP5), which delivers RA from the cytosol to nuclear PPAR*β*/*δ* and cellular RA-binding protein II (CRABP-II) which shuttles it to RAR [7, 8, 14–18]. In accordance, it has been shown that RA inhibits the growth of mammary carcinomas that express a high CRABP-II/FABP5 ratio but facilitates proliferation of mammary carcinomas in which this ratio is low [5, 13, 15, 19]. 

FABP5 and its cognate receptor PPAR*β*/*δ* thus appear to function as oncogenes, and it has been suggested that inhibition of their transcriptional activities may comprise a novel strategy for treatment of some cancers [12, 13]. 

A question that arises from these observations is whether activation of the FABP5/PPAR*β*/*δ* path underlies tumor development in cancers other than specific breast cancers. In support of this notion, it has been reported that the expression of FABP5 is upregulated in carcinomas of the pancreas, breast, bladder, and prostate [20]. It was also reported that FABP5 can serve as a prognostic marker and that it induces metastasis of prostate cancer [20, 21]. This activity could be traced to the ability of FABP5 to upregulate the expression of VEGF [22, 23]. 

 The present study was undertaken in order to examine whether FABP5 and PPAR*β*/*δ* are involved in regulation of prostate cancer cell growth and to obtain insight into mechanisms by which the expression of FABP5 is regulated. We show that PPAR*β*/*δ* directly induces the expression of FABP5 through a functional PPRE within the promoter region of the FABP5 gene. We show further that activation of the FABP5/PPAR*β*/*δ* pathway enhances the proliferation of malignant prostate cancer cell line PC3M, and that downregulation of either protein inhibits the growth of these cells. The data reveal the existence of a positive feedback loop that enhances the transcriptional activities of the FABP5/PPAR*β*/*δ* pathway in prostate cancer cells, and they indicate that these activities support prostate carcinoma cell proliferation and tumorigenicity.

## 2. Results

### 2.1. Prostate Cancer Progression Is Accompanied by Upregulation of FABP5/PPAR*β*/*δ* Expression and Signalling

**Figure 1:**
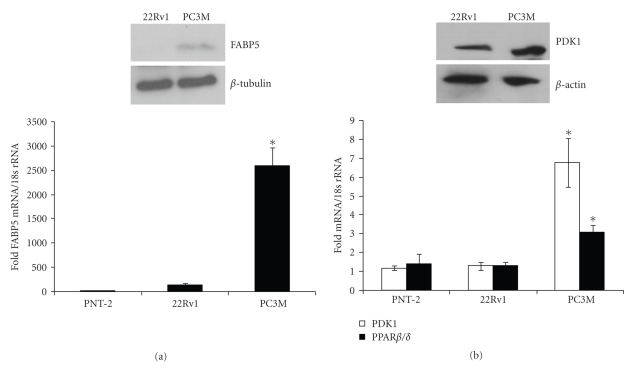
Prostate cancer progression is accompanied by up regulation of FABP5/PPAR*β*/*δ* expression and signalling. (a) Bottom: Expression levels of FABP5 mRNA in denoted cell lines were measured by Q-PCR. Top: Level of FABP5 protein in denoted cell lines assessed by immunoblots. (b) Bottom: Expression levels of PDK-1 and PPAR*β*/*δ* mRNA in denoted cell lines were measured by Q-PCR. Top: Immunoblots of PDK1 in denoted cell lines. Data are mean ± S.D. (*n* = 3). *P < .02 versus 22Rv.1. (Paired *T* test). Immunoblots were repeated three times with similar results.

To begin to examine whether FABP5 and PPAR*β*/*δ* may play a role in prostate cancer cell growth, the expression levels of these genes were assessed in three prostate cancer cell lines, the benign PNT-2 cells, the mildly oncogenic 22Rv1 cells, and the highly malignant PC3M cells [24–27]. The levels of FABP5 and PPAR*β*/*δ* mRNA were measured by quantitative real-time PCR (Q-PCR) and FABP5 protein expression was assessed by immunoblots. The data (Figures 1(a) and 1(b)) indicated that expression of both *FABP5* and *PPAR* *β*/*δ* markedly higher in PC-3M cells. Correspondingly, expression of the direct PPAR*β*/*δ* target gene PDK1 [28], a kinase involved in activation of survival pathways, was also elevated in PC-3M cells (Figure 1(b)). Hence, the expression as well as the transcriptional activity of the FABP5/PPAR*β*/*δ* pathway appear to correlate with the tumorigenic potential of these cell lines.

### 2.2. FABP5 Is a Direct Target Gene for PPAR*β*/*δ*

**Figure 2:**
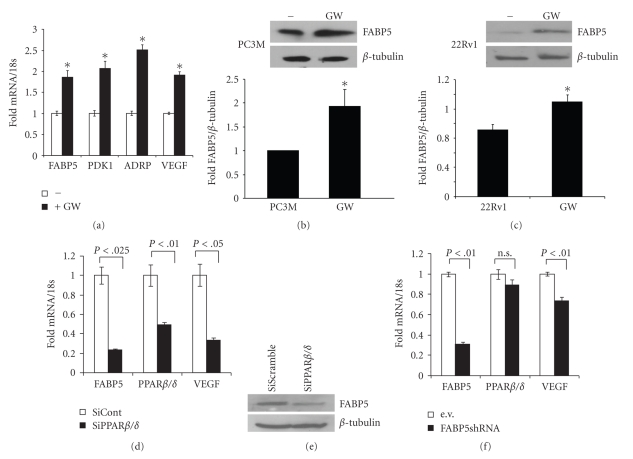
Activation of PPAR*β*/*δ* results in upregulation of FABP5. (a) PC3M cells were treated with GW0742 (1 *μ*M, 4 h). Expression of FABP5, PDK1, ADRP, and VEGF were assessed by Q-PCR. The data were normalized to the untreated control. (b, c) Denoted cells were treated with 1 *μ*M (a) or 2 *μ*M (b) GW0742 for 16 h. Left panels, immunoblots of FABP5 in untreated versus GW0742-treated cells. Right panel: Densitometry analyses depicting changes in FABP5 expression upon treatment with GW0742 in three independent experiments (mean ± S.D.). (d, e) PC3M cells were transfected with vectors harboring SiScramble or PPAR*β*/*δ* siRNA. Three days later, expression of FABP5, PPAR*β*/*δ*, and VEGF mRNA in were measured by Q-PCR (d) and FABP5 expression was assessed by immunoblot (e). (f) PC3M cells were stably transfected with an empty vector (PGIPZ), or a vector harbouring shFABP5. Expression levels of FABP5, PPAR*β*/*δ*, and VEGF mRNA were measured by Q-PCR. *P < .05 versus nontreated controls.

To examine whether the expression of FABP5 may be controlled by PPAR*β*/*δ*, PC3M cells were treated with the synthetic PPAR*β*/*δ*-selective ligand GW0742. Effects of the treatment on the expression of two known PPAR*β*/*δ* target genes, PDK1 and adipose differentiation-related protein (ADRP [29]), and on the expression of FABP5 was assessed by Q-PCR. GW0742 upregulated the expression of PDK1 and ADRP in these cells (Figure 2(a)). Similarly to the response of these direct PPAR*β*/*δ* target genes, GW0742 treatment also resulted in upregulation of FABP5 mRNA (Figure 2(a)) and protein (Figure 2(b)). A similar response was observed in 22Rv1 cells (Figure 2(c)). Decreasing the expression level of PPAR*β*/*δ* by transfecting PC3M cells with an expression vector harbouring siRNA towards the receptor downregulated the expression of the direct PPAR*β*/*δ* target gene VEGF as well as the expression of FABP5 (Figures 2(d) and 2(e)). In accordance with the known function of FABP5 in supporting PPAR*β*/*δ* function, decreasing the expression of the binding protein using a lentoviral vector encoding FABP5shRNA downregulated the expression of VEGF (Figure 2(f)). 

**Figure 3:**
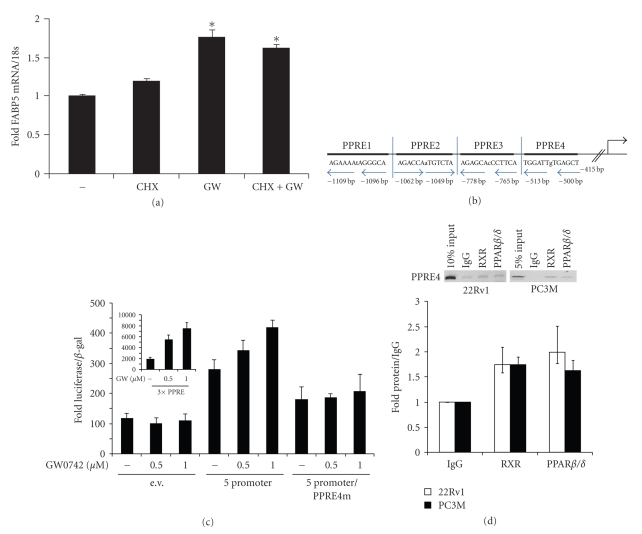
FABP5 is a direct target gene for PPAR*β*/*δ*. (a) PC3M cells were treated with cycloheximde (20 *μ*g/mL) for 10 min prior to addition of GW0742 (1 *μ*M, 4 h). Expression of FABP5 mRNA was measured by Q-PCR. Data are mean ± S.D. (*n* = 3). *P < .05 versus nontreated control. (b) Location of putative PPREs in the FABP5 promoter. (c) Transcriptional activation assays utilizing a luciferase reporter driven by a 900 bp region of the FABP5 promoter which encompasses the four putative PPREs (5 promoter). In the mutant reporter construct (5 promoter/PPRE4m), the AGCTCA sequence in PPRE4 was exchanged to AGCTTT. PC3M cells were cotransfected with the denoted reporter and an expression vector for *β*-galactosidase. 24 h later, cells were treated with vehicle or GW0742 and cultured for 24 hr. Luciferase activity was measured and normalized to the activitiy of b-galactosidase. Data are mean ± S.D. (*n* = 3). Inset: Luciferase assays carried out with a luciferase reporter driven by 3 consensus PPREs. (d) Chromatin immunoprecipitation assays in denoted cell lines. Immunoprecipitations were carried out using denoted antibodies and the PPRE4 region of the FABP5 promoter amplified by PCR. Bottom panel: quantification of 3 independent ChiP assays (mean ± SEM).

To examine whether FABP5 is a direct target for PPAR*β*/*δ* or whether the response reflects secondary events, the effect of the PPAR*β*/*δ*-selective ligand GW0742 on FABP5 expression was examined in the presence of the protein synthesis inhibitor cycloheximide. PC3M cells were pretreated with vehicle or cycloheximide for 10 min prior to addition of the GW0742. Cells were incubated for 4 hr. and the level of FABP5 mRNA measured by Q-PCR. GW0742 induced the expression of FABP5 both in the absence and in the presence of cycloheximide (Figure 3(a)), indicating that the effect did not require *de novo* protein synthesis raises the possibility that FABP5 is a direct target gene for PPAR*β*/*δ* in PC3M prostate cancer cells.

### 2.3. The FABP-5 Promoter Contains a Functional PPRE

The software Nubiscan (http://www.nubiscan.unibas.ch/) was used to identify PPAR response elements (PPRE) that may mediate the ability of PPAR*β*/*δ* to upregulate FABP5. Four potential PPREs were identified within 900 bp upstream from the FABP5 transcription start site (Figure 3(b), PPRE1-PPRE4). The region was cloned into a luciferase reporter construct, and transcriptional activation assays were carried out. PC3M cells were transfected with a luciferase reporter driven by the minimal prolactin promoter (prl-luc) or a luciferase reporter containing the 900 bp sequence of the FABP5 promoter (FABP5-luc). Cells were also cotransfected with an expression vector for PPAR*β*/*δ* and with a plasmid encoding *β*-galactosidase, which served as a transfection control. Cells were treated with vehicle or GW0742 for 24 hr, lysed, and lysates assayed for luciferase activity. The data (Figure 3(c)) show that the 900 bp promoter region of FABP5 enhanced the basal activity of the reporter as well as responded to treatment with GW0742 in a dose-dependent fashion, indicating the presence of a functional PPRE. To more precisely localize the PPRE, transcriptional activation assays were carried out utilizing reporter constructs in which the putative response elements were individually mutated. Mutation of the most proximal element, PPRE4, abolished the ability of the reporter to respond to the ligand (Figure 3(c)), indicating that the element is necessary of PPAR*β*/*δ*-mediated upregulation of FABP5. In agreement, chromatin immunoprecipitation assays (ChIP) indicated that both PPAR*β*/*δ* and its heterodimerization partner RXR are recruited to PPRE4 (but not the other putative response elements) in both 22Rv1 and PC3M cells (Figure 3(d)). Taken together, the data demonstrate that PPAR*β*/*δ* directly induces the expression of its cognate intracellular lipid binding protein FABP5.

### 2.4. The FABP5/PPAR*β*/*δ* Pathway Enhances Proliferation of PC3M Prostate Cancer Cells

**Figure 4:**
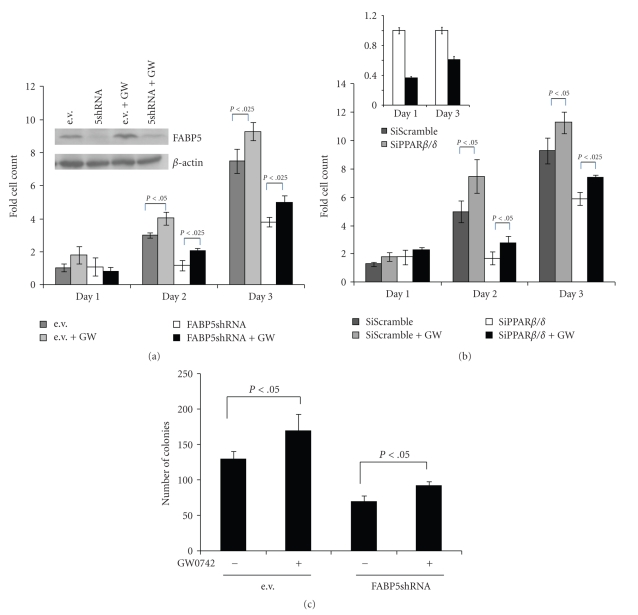
The FABP5/PPAR*β*/*δ* pathway enhances prostate cancer cell proliferation and transformation. (a) PC3M cells that stably express shFABP5 were cultured in a 24 well plate (2500 cells/well) and treated with vehicle or GW 0742 (1 *μ*M). Cells were counted at the denoted days. Data are mean ± SEM (*n* = 3). Inset: immunoblotting demonstrating low FABP5 level in shFABP5-expressing. (b) PC3M cells were transfected with a vector harboring PPAR*β*/*δ*siRNA. Four days later, were cultured in a 24 well plate (2500 cells/well) and treated with vehicle or GW 0742 (1 *μ*M). Cells were counted at the denoted days. Inset: Q-PCR analyses demonstrating low PPAR*β*/*δ* in cells transfected with SiRNA towards the receptor. (c) Colony formation assays were conducted in the absence or presence of GW0742 (1 *μ*M). Cells were cultured in 2% agarose for 29 days (see Materials and methods for details). Media was replenished every 4 days. Colonies were visualized by staining with 0.005% crystal violet and counted under a light microscope. Data are mean ± SEM (*n* = 3).

FABP5 and PPAR*β*/*δ* is involved in enhancing cell proliferation and survival in keratinocytes and some mammary carcinoma cells [5, 13, 30]. To examine the involvement of the pathway in regulation of prostate cancer cell growth, the effect of treatment of PC3M cells with GW0742 was examined. The data (Figure 4(a)) showed that GW0742 markedly enhanced cell proliferation as early as day 2. The PPAR*β*/*δ*-selective ligand also increased the proliferation of the less malignant 22Rv1 (data not shown). Strikingly, reducing the expression level of either FABP5 (Figure 4(a)) or PPAR*β*/*δ* (Figure 4(b)) markedly retarded cell proliferation as well as hampered the proliferative response to GW0742. To monitor effect of the FABP5/PPAR*β*/*δ* pathway on the ability of PC3M cells to form colonies in a sub strata-free environment, an established hallmark of tranformation, cells that stably express scrambled shRNA or shRNA towards FABP5 were generated and colony formation assays were carried out in the absence or presence of GW0742 (Figure 4(c)). The data demonstrated that GW0742 significantly increased the number of colonies, and that downregulation of FABP5 inhibited colony formation both in the absence and presence of ligand.

## 3. Discussion

**Figure 5:**
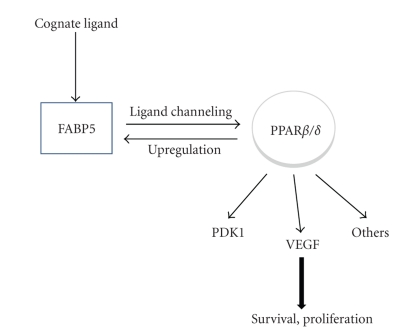
A model for the cooperation between FABP5 and PPAR*β*/*δ* which enhances prostate cell survival and proliferation. Upon binding to a cognate ligand, FABP5 translocates to the nucleus where it directly delivers the ligand to its cognate nuclear receptor, PPAR*β*/*δ*. Activation of PPAR*β*/*δ* results in upregulation of FABP5. A positive feedback loop is thus established: PPAR*β*/*δ* activation induces the expression of FABP5 which, in turn, enhances the transcriptional activity of the receptor. The FABP5/PPAR*β*/*δ* pathway induces the expression of PPAR*β*/*δ* target genes involved in cell survival, for example, PDK1, and growth and angiogenesis, for example, VEGF, and thus contributes to prostate cancer development.

The data presented above demonstrate that FABP5, which functions to selectively deliver ligands to the nuclear receptor PPAR*β*/*δ*, is under the direct control of its cognate nuclear receptor in prostate cancer cells. In the presence of an activating ligand, FABP5 is imported into the nucleus where it directly “channels” the ligand to PPAR*β*/*δ* [5, 18]. In turn, in prostate cancer cells, activated PPAR*β*/*δ* upregulates the expression of FABP5. The resulting positive-feedback loop augments the overall activity of the FABP5/PPAR*β*/*δ* pathway, leading to efficient induction of PPAR*β*/*δ* target genes involved in cell proliferation and survival and supporting prostate cancer cell growth (Figure 5). Hence, similarly to its ability to enhance the proliferation of keratinocytes [5, 11, 30] and to drive mammary tumor growth in the breast cancer mouse model MMTV-*neu* [5, 13], the FABP5/PPAR*β*/*δ* pathway induces prostate cancer cell growth (Figure 4). These data suggest that antagonists for FABP5 or PPAR*β*/*δ* may be efficacious therapy and perhaps prevention of some cancers. The identification of a selective antagonist for PPAR*β*/*δ* has been reported [31] but, to the best of our knowledge, the ability of the compound to inhibit cancer cell growth has not yet been examined. No antagonist for FABP5 is currently available. However, a synthetic inhibitor for the related protein FABP4 has been developed and was shown to improve insulin resistance and atherosclerosis in mice [32]. Intracellular lipid-binding proteins may thus be targeted for therapy and prevention of disease. Efforts to identify a small molecule inhibitor for FABP5 are ongoing.

Of the fourteen known intracellular lipid binding proteins (iLBPs), four have been shown to cooperate with specific nuclear receptors. It was reported that CRABP-II delivers RA to RAR, and that FABP1, FABP4, and FABP5 shuttle cognate ligands to PPAR*α*, PPAR*γ*, and PPAR*β*/*δ*, respectively [7, 8, 14–18, 33, 34]. Cooperation of iLBPs with nuclear receptors requires that the binding proteins be able to move in and out of the nucleus and that they do so in a ligand-dependent manner. Indeed, a ligand-controlled nuclear localization signal has been identified in CRABP-II [17] and FABP4 [35] and a constitutive nuclear export signal was found in FABP4 [35]. Sequence homology considerations suggest that such signals are present in some but not all ILBPs. Hence, some iLBPs may play roles outside the nucleus and that their functions may be unrelated to those of nuclear receptors. It is interesting to note that the promoters of three of the iLBPs known to function in conjunction with nuclear receptors harbor response elements for their cognate receptors. Thus, the CRABP-II promoter harbors a consensus RARE [36], the expression of FABP4 is directly regulated by PPAR*γ* in adipocytes [37], and, as shown here, FABP5 is a direct target gene for PPAR*β*/*δ* in prostate cancer cells. The ability of these receptors to induce the expression of their binding proteins is however cell-specific. For example, while RAR upregulates CRABP-II in human skin fibroblasts [36], it does not induce the expression of the protein in rat uterus [38]. Similarly, activated PPAR*β*/*δ* effectively upregulates FABP5 expression in prostate cancer cells (Figure 2) but has little effect on the expression of the gene in adipocytes (our unpublished observations). The basis for the cell specificity of the feedback-loop between binding proteins and their cognate nuclear receptor remains to be clarified.

## 4. Materials and Methods

### 4.1. Reagents

Plasmid harbouring FABP5shRNA was obtained from Open Biosystems (172-0471-C-3 — V2LHS-131713). Oligonucleotides for siPPAR*β*/*δ* were from Applied biosystems). Transfections were performed using superfect transfection reagent as per manufacturers' protocol. Antibodies against RXR (sc-774) and PPAR-*β*/*δ* (sc-7197), rabbit IgG (sc-2027), and protein A/G agarose (sc-2003) were purchased from Santa Cruz Biotechnology Inc. Antibodies against FABP5 (AF3007) and PDK1 (61107) were obtained from R&D Systems and Transduction Laboratories, respectively.

### 4.2. Cells

PC3M and 22Rv1 cells were cultured in L-Glutamine containing RPMI medium supplemented with 10% fetal calf serum, penicillin (100 U/ml), and streptomycin (100 mg/ml) (Invitrogen Life Sciences, Carlsbad, CA). Prior to treatment with ligands, cells were grown in medium containing 10% charcoal treated serum for 12 hr. Depending on whether mRNA or protein analysis was to be performed, cells were exposed to ligands for 4 hr or 16 hr, respectively. In proliferation, colony formation, wound healing assays, and ligand supplementation were replenished every 24 hr.

### 4.3. Bioinformatics

Primers were designed using the primer3 primer design software (http://frodo.wi.mit.edu/). Primer selectivity was validated using a nucleotide blast program (http://blast.ncbi.nlm.nih.gov/Blast) and primers purchased from Integrated DNA Technologies (Coralville, IA).

### 4.4. Immunoblots

Total cell protein was extracted using RIPA buffer (25 mM Tris-HCl, 150 mM NaCl, 1% NP40, 1% sodium deoxycholate, and 0.1% SDS). Proteins were resolved by electrophoresis on 10% SDS-PAGE gels and transferred onto Immobilon-P^sq^ membrane (Millipore). Membranes were incubated with primary antibodies (1 hr), followed by 3 washes with Tween-TBS, and incubation with HRP conjugated antibodies. Protein expression was detected by exposure to ECL and exposed to XR-B x-ray film. Band intensities were quantified using AlphaImager 2000 software (Alpha Innotech).

### 4.5. Transcriptional Activation Assays

To generate reporter plasmids, the promoter of FABP-5 containing the identified putative PPREs was amplified by PCR and KpnI and MluI restriction sites overhangs were added using the primers 5′-CCGGGTACCTCAGAGCTCCATCACAGCTAC-3′ (sense) and 5′-CCGACGCGTACTGCAGGAAGTGATTGATCG-3′ (antisense). PCR products were integrated into an pRL-PGL3 reporter vector. The pRL-PGL3 vector was transformed into XL1 Blue competent cells, colonies selected, amplified and verified by DNA sequencing. PC3M cells were transfected with pRL-PGL3 reporter vector, a plasmid encoding *β*-galactosidase, and expression vectors for PPAR*α*, *β*, or *γ*. Cells were treated with ligands for 24 hrs, lysed and luciferase activity measured using the luciferase assay system. Luciferase activity was normalized to *β*-galactosidase activity.

### 4.6. Quantitative Real-Time PCR

Total RNA was extracted using Trizol (Invitrogen), and cDNA generated using a high capacity cDNA reverse transcription kit (Applied Biosystems). mRNA levels were measured by Q-PCR using Taqman chemistry and assays on demand probes (Applied Biosystems) for FABP5 (Hs2339439_g1), ADRP (Hs00765634_m1), PDK1 (Hs01561850_m1), PPAR*β*/*δ* (Hs00606407_m1), and VEGF (Hs00173626_m1). 18s ribosomal RNA was used as a loading control.

### 4.7. Chromatin Immunoprecipitation

PC3M cells were grown to 80% confluency in charcoal treated serum for 24 hr prior to ligand treatment. Proteins were crosslinked to DNA using 10% formaldehyde (20 min, 37°C). Reaction was stopped with glycine (125 mM, 5 min 4°C), cells were washed three times in ice cold PBS, scraped, lysed (1% SDS, 10 mmol/L EDTA, 50 mM, Tris at pH 7.9, 1 mM DTT, leupeptin, pepstatin A, and aprotinin), incubated (4°C, 30 min), and sonicated. One fifth of the sample was stored as input, whereas the remaining samples were diluted with 0.5% Triton X-100, 2 mMEDTA, 2 mM Tris (pH 7.9), 150 mM NaCl, 1 mM DTT, protease inhibitors, and salmon sperm DNA, and precleared (1 hr, 4°C) with protein A beads. Following centrifugation, supernatants were incubated with appropriate antibodies overnight and protein A beads were added (2 hr). Beads were washed with 0.25% NP40, 0.05% SDS, 2 mM EDTA, 20 mM Tris (pH 8), 250 mM NaCl and protease inhibitors and with TE buffer, re-suspended in NaHCO_3_ containing 1% SDS, and the crosslink reversed (12 hr, 65°C). Proteins were degraded by proteinase K (80 *μ*g/ml, 1 hr), DNA purified by phenol-chloroform extraction and appropriate regions amplified by PCR. PCR primers for PPRE4: sense, 5′-CCCAACAGGGATAAAATCCT-3′; antisense, 5′-TACCCGGACCAATCGATTA-3′.

### 4.8. Colony Formation Assays

A basement layer of a 1 : 1 mixture of 2% agarose in Tris-borate buffer and RPMI 1640 medium was cast in a 6-well plate at 4°C. Cells were suspended in the same agarose-RPMI mixture at room temperature at a density of 5 × 10^3^ cells/ml. The mixture was decanted onto the preset basement gel layer and incubated at 4°C to solidify. Once set, the cells in the plates were grown for 29 days. Media were replenished every 4 days. To visualize colonies, cells were stained with 0.005% crystal violet and counted under a light microscope.
